# The Role of Transabdominal Ultrasound Elastography in Gastrointestinal Non-Liver Diseases: Current Application and Future Prospectives

**DOI:** 10.3390/diagnostics13132266

**Published:** 2023-07-04

**Authors:** Mattia Paratore, Matteo Garcovich, Maria Elena Ainora, Livio Enrico Del Vecchio, Giuseppe Cuccia, Laura Riccardi, Maurizio Pompili, Antonio Gasbarrini, Maria Assunta Zocco

**Affiliations:** 1 Medicina Interna e Gastroenterologia, CEMAD Digestive Disease Center, Fondazione Policlinico Universitario A. Gemelli IRCCS, Largo A. Gemelli 8, 00168 Rome, Italy; mattia.paratore01@gmail.com (M.P.); matteo.garcovich@policlinicogemelli.it (M.G.); mariaelena.ainora@policlinicogemelli.it (M.E.A.); livioenricodelvecchio@gmail.com (L.E.D.V.); giuseppecuccia24@gmail.com (G.C.); laura.riccardi@policlinicogemelli.it (L.R.); maurizio.pompili@unicatt.it (M.P.);; 2 Medicina Interna e del Trapianto di Fegato, Fondazione Policlinico Universitario A. Gemelli IRCCS, Largo A. Gemelli 8, 00168 Rome, Italy; 3 Università Cattolica del Sacro Cuore, Largo A. Gemelli 8, 00168 Rome, Italy

**Keywords:** elastography, gastrointestinal diseases, shear wave elastography, liver stiffness, inflammatory bowel disease, ultrasound

## Abstract

Ultrasound imaging is the first-line investigation for patients with abdominal symptoms, as it effectively depicts the gastrointestinal tract and enables the diagnosis of multiple pathological conditions. Among different recent ultrasound technological advancements, elastography enables the evaluation of various tissue characteristics, such as neoplastic transformation or fibroinflammatory status. In recent years, ultrasound elastography has been utilized extensively for the study of liver diseases and in numerous other clinical settings, including gastrointestinal diseases. Current guidelines suggest the use of transabdominal ultrasound elastography to characterize bowel wall lesions, to assess gastrointestinal contractility, to diagnose and grade chronic pancreatitis; however, no specific indications are provided. In the present paper, we summarize the evidence concerning the application of different ultrasound elastography modalities in gastrointestinal non-liver diseases.

## 1. Introduction

Ultrasound (US) imaging is the first-line investigation for patients with abdominal symptoms, as it effectively depicts the gastrointestinal tract and enables the diagnosis of multiple pathological conditions [1]. Recent technological advancements have resulted in the development of several kinds of US-based methodologies that enable the evaluation of various tissue characteristics. The term “multiparametric ultrasound” refers to the application of all these techniques to achieve the diagnosis [2,3]. Among these, elastography is one of the most promising.

Elasticity is a property of a tissue to resist deformation and revert to its original shape after being subjected to a stress. Neglecting the viscous component, elastic properties of a tissue can be described according to Hooke’s law (Equation (1)):

(1) $$E=\frac{\sigma }{\varepsilon }$$

where *σ* is the stress applied, *ε* is the strain produced, and *E* is the elastic modulus or Young’s modulus. High Young’s modulus indicates that the applied stress results in less strain; consequently, the tissue has low elasticity (or high stiffness), and vice versa. When shear stress produces a shear strain tangential to the surface, elastic modulus is indicated as shear modulus (*G*) [4].

Shear modulus is related to speed propagation of shear waves in a medium and to Young’s modulus. Equation (2) described this relationship under the assumption that for incompressible medium, the Poisson’s ratio (*v*) is approximatively 0.5:

(2) $$E=2\left(v+1\right)G=3G=3\rho c_{s}^{\;2}$$

where *G* is the shear modulus, *c_s_* is the shear wave speed, and ρ is the medium density. Speed of shear waves is higher in medium with higher stiffness [5]. Thus, elastography techniques can be divided into two categories: strain elastography (SE) and shear wave elastography (SWE). The SE is based on Young’s modulus estimation; strain is induced manually applying pressure or using acoustic radiation force impulse (ARFI). Due to the inability to assess the stress distribution, the SE provides a qualitative (i.e., colour-based classification) or semi-quantitative (i.e., strain ratio, scores) assessment of the examined tissue. Strain ratio is the ratio between normal (high strain, low stiffness) and pathological (low strain, high stiffness) tissue. Thus, it is higher in high-stiffness tissue [6].

In the SWE, the study of shear waves properties is used to calculate shear modulus or shear wave velocity and estimate tissue stiffness. Shear waves can be generated by mechanical vibration in one-dimensional or two-dimensional transient elastography or by an ARFI in point- and two-dimensional shear wave elastography (p-SWE and 2D-SWE, respectively). The results of SWE can be displayed as either shear wave speed (m/s) or shear or Young’s modulus (kPa) due to the relation expressed in Equation (2) [5,7], but elasticity scale allows for more discrimination between stiffness values [8].

In pathological phenomena such as neoplastic transformation and fibroinflammatory status, the elastic properties of tissue change and elastography could theoretically be a reliable method for detecting and quantifying these changes [9,10].

After its development in the 1990s [11], early applications of US elastography included the study of neoplastic lesions and fibroinflammatory status in easily accessible tissues, such as the breast, prostate and liver tissues [12,13]. In more recent times, US elastography has been utilised extensively in numerous clinical settings, including gastroenterology, but not limited to the study of liver diseases.

Current guidelines suggest the use of transabdominal ultrasound elastography (US-E) to characterise bowel wall lesions, assess gastrointestinal contractility, diagnose and grade chronic pancreatitis (CP); however, no specific indications are provided [14,15].

In the present paper, we summarize the evidence concerning the application of different US-E modalities in gastrointestinal non-liver diseases.

## 2. Upper Gastrointestinal Tract

Few studies employed the US-E to evaluate the relationship between esophagogastric wall elasticity and motility, and only two case reports describe the US-E characteristics of gastric wall neoplasia.

Real-time SE using the cardiovascular pulsation as pressure was used to count the change in stiffness (CS) of esophagogastric junction (EGJ) for ≥15 s in patients undergoing esophagogastroduodenoscopy. The number of CS was significantly lower in patients with reflux esophagitis compared to patients with non-reflux esophagitis and in multivariate analysis; the absence or presence of reflux esophagitis was the only significant factor related to CS. Furthermore, CS showed an area under the curve (AUC) of 0.84 for the diagnosis of reflux esophagitis. These results suggest that US-E might be useful to assess the EGJ function [16]. In another study, the US-E was used to assess the oesophagus involvement in systemic sclerosis (SSc). Particularly, the 2D-SWE of the abdominal oesophagus and EGJ showed no difference between patients with SSc and controls, but interestingly, after drinking water, the decrease in the SWE values was greater for controls than for patients with SSc, suggesting a reduced elasticity in the SSc group [17]. Using a specific method called strain rate imaging [18], Ahmed et al., demonstrated that US-E can distinguish specific subtypes of functional dysplasia, supporting the clinical Rome III classification [19]. Regarding neoplastic disorders, two case reports described the US-E features of large gastric cancer and gastrointestinal stromal tumour, respectively. In both lesions, the stiffness was increased [20,21].

Further studies are needed to establish the role of US-E in assessing the organic lesions and motility disorders of the upper gastrointestinal tract.

## 3. Lower Gastrointestinal Tract

**Figure 1:**
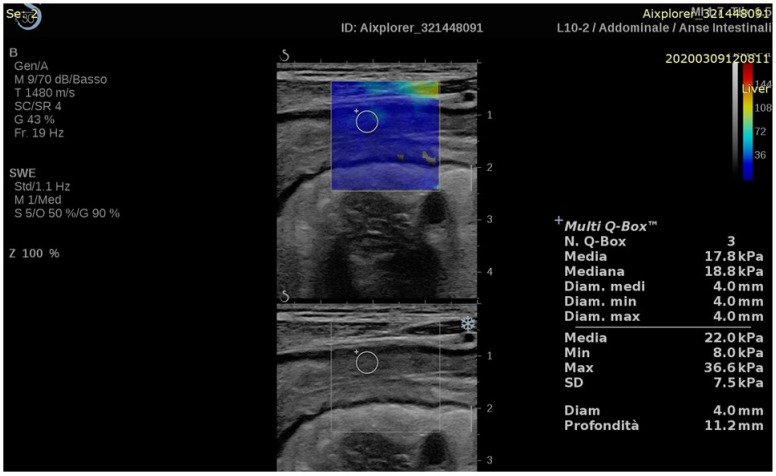
Longitudinal 2D-shear wave elastography (upper part of the image) and corresponding B-mode (lower part of the image) of the terminal ileum measured with SSI.

Despite the methodological limits imposed by the normal anatomy and physiology of the lower gastrointestinal tract [22], one of the most relevant application of US-E entails the non-invasive assessment of chronic inflammatory bowel disease (IBD). Studies concerning the use of the US-E in IBD are listed in Table 1, while an example image of US-E of the intestinal wall is depicted in Figure 1.

US-E showed a practical usefulness also in the emergency setting of acute appendicitis, while no studies investigated the role of US-E in patients with other common acute intestinal disorders such as diverticulitis.

### 3.1. Inflammatory Bowel Disease

The IBD are characterized by chronic intestinal wall inflammation caused by immunoinflammatory altered mechanisms [39]. Despite the fact that Crohn’s disease (CD) and ulcerative colitis (UC) share numerous pathogenetic mechanisms, their histopathological and clinical features are distinct. In CD, inflammation involves the entire intestinal wall and is more closely associated with fibrotic progression than in UC, where inflammation involves only the mucosal and submucosal layers of the intestinal wall. Moreover, CD can affect all digestive tracts, but it is most prevalent in the terminal ileum, whereas UC is restricted to the colon and ileocecal valve [40,41]. Considering these assumptions, it is understandable why the US-E has been investigated substantially more in the CD than the UC.

#### 3.1.1. US-E, Disease Activity and Fibro-Inflammatory Characterization in Crohn’s Disease

Normal stiffness of terminal ileum in 139 healthy volunteers was described by Zhao et al., using 2D-SWE. The mean 2D-SWE value was 1.08 ± 0.25 m/s (or 3.84 ± 1.84 kPa). Different physiological factors such as gender, age, body mass index (BMI), profundity and bowel wall thickness did not appear to significantly influence these values [42]. Higher-than-normal p-SWE values were observed in two cohorts of apparently “normal” terminal ileum and sigma of CD patients; however, these values were lower than those in inflamed ileum and sigma (1.42 vs. 2.73 m/s in the ileum and 1.81 vs. 2.22 m/s in the sigma) [30]. This suggests that patients with CD have increased bowel wall stiffness outside of acute inflammatory episodes, and that this stiffness increases during disease flare-ups.

As only the inflammatory form is medically treatable, the most investigated applications of US-E in IBD concern its ability to differentiate between inflammatory and fibrotic bowel lesions.

In an ex vivo human study analysing 17 bowel segments resected for known or suspected IBD, Dillman et al., demonstrated that the p-SWE values were significantly higher in high-fibrosis segments compared to low-fibrosis ones, whereas there was no difference between high-inflammation and low-inflammation segments. Furthermore, the p-SWE results moderately correlated with the fibrosis score but not correlated with inflammation [43]. In another study, Baumgart et al., demonstrated that real-time SE values measured in vivo were significantly correlated with the fibrosis score and tensiometer-measured strain of surgically resected tissue [44]. Thus, elastography seem to be effectively related to bowel fibrosis.

Different studies assessed the association between fibrosis and qualitative, semi-quantitative and quantitative US-E. A semi-quantitative SE-based score was lower for inflammatory stenosis than for fibrotic stenosis, indicating that fibrotic stenosis is significantly stiffer [35]. The strain ratio increased with fibrosis progression and showed an excellent accuracy to diagnose severe fibrosis (AUC of 0.91). In addition, fibrosis was the only factor independently associated with strain ratio [36]. On the contrary, Serra et al., found no significant correlation between the strain ratio and histologically assessed fibrosis, inflammatory scores and clinically and biochemically assessed disease activity [34].

Recent studies have expanded the evidence regarding the role of SWE in IBD assessment. In a prospective study including 35 patients undergoing resection within one week after US-E, the 2D-SWE results were significantly higher in severe fibrosis compared to moderate and mild fibrosis (23 ± 6.3 vs. 17.4 ± 3.8 and 14.4 ± 2.1 kPa). A cut-off value of 22.6 kPa distinguished severe from mild/moderate fibrosis with high accuracy (AUC, sensitivity and specificity of 0.82, 70% and 91%, respectively). However, no differences were observed between consecutive inflammation grades [29]. Compared to SE, the p-SWE showed a better performance in predicting predominantly inflammatory or fibrotic strictures with an accuracy, specificity, sensitivity, positive and negative predictive values of 96%, 100%, 75%, 100% and 95.5% respectively, using a cut-off value of 2.73 m/s [27].

Since inflammatory and fibrotic tissue have distinct vascularization, a combination of US techniques could further differentiate the two categories. Inflammatory and fibrotic bowel stenosis showed differences in both the 2D-SWE and contrast-enhanced US (CEUS) parameters (peak enhancement, time to peak and AUC) [26]. In addition, the p-SWE showed a negative moderate correlation with peak enhancement [33]. Confirming this, a combined score based on real-time SE, B-mode and CEUS showed a higher accuracy to distinguish inflammatory and fibrotic tissue than each single technique [32].

#### 3.1.2. US-E in Ulcerative Colitis

Only few studies, as far as we are aware, has examined patients with UC. Ishikawa et al., were the first to demonstrate alterations in SE related to colonoscopy findings and disease activity in UC [38]. Subsequently, Goertz et al., compared the p-SWE values measured in all colon segments of twenty patients with UC to those of thirteen healthy controls. The p-SWE values for the transverse and sigmoid colons were significantly higher in patients with UC compared to controls (1.94 vs. 1.55 m/s, *p* = 0.045 and 2.18 vs. 1.76 m/s, *p* = 0.032, respectively). Mayo subscore and C-reactive protein (CRP) levels were not significantly correlated with the p-SWE values within the colon segments, indicating that disease activity is not related to the SWE values [28]. Similarly, in a prospective study conducted by Yamada et al., 26 patients with UC who underwent colonoscopy were enrolled. The 2D-SWE of the sigmoid-colon performed 48 h before or after colonoscopy had significantly higher results in the mucosal healing group than in the active phase group (2.4 vs. 1.62 m/s, *p* = 0.007) and negatively correlated with clinical severity score [25]. In active UC, oedema, erosion, crypt abscess and necrosis may cause tissue softness.

#### 3.1.3. US-E and IBD in Paediatric Population

In paediatric patients with CD, Fufezan et al., demonstrated that US-E findings were related to disease activity. A qualitative and semi-quantitative evaluation of 48 bowel segments based on the SE colour code and strain ratio was performed. Both methods showed a significant correlation with fat infiltration and disease activity markers [37]. Recently, using histology on ileal resected strictures as a reference method, Abu-Ata et al., showed a significant association between the values of the 2D-SWE of bowel wall and the entire elastogram of the right lower abdominal quadrant and mucosal inflammation and muscularis mucosa inner layer’s smooth muscle hypertrophy, while no correlation was demonstrated with fibrosis [23].

#### 3.1.4. US-E and IBD Treatment

Several therapeutic alternatives are currently available for IBD. Among them, antitumour necrosis factor (TNF) or anti-interleukins is widely used in moderate-to-severe disease [45,46]. US-E could provide prognostic information regarding the response to treatment and its management.

In a prospective study conducted by Orlando et al., the strain ratio at baseline and 14 and 52 weeks after starting anti-TNF therapy was evaluated. The strain ratio at baseline was significantly higher in patients who underwent surgery during the follow-up compared to the non-surgical group (2.22 vs. 1.48, *p* = 0.009) and was significantly lower in patients who achieved mucosal healing (defined as bowel wall thickness ≤ 3.0 mm) after 14 week of anti-TNF therapy compared to patients who did not achieve this outcome (1.06 vs. 1.58, *p* = 0.03) [31]. In contrast, Chen et al., evaluating response to therapy at 14 weeks with clinical and endoscopic assessments, found that the 2D-SWE values decreased substantially after 14 weeks in patients who responded to therapy, suggesting the use of SWE for the early assessment of response to therapy [24]. Further research and validations using different biological therapies and larger sample sizes are required to validate the clinical utility of US-E, perhaps including the use of multiparametric US to optimise non-invasive strategies [47].

### 3.2. Appendicitis

Multiple studies demonstrated that US-E of appendix differs significantly between patients with acute appendicitis (AA) and healthy controls.

In a prospective study including 11 healthy volunteers and 41 patients with suspected AA, the 2D-SWE had higher results in definitive AA cases compared to patients with suspected but unconfirmed AA and controls (25 vs. 10.4 and 8.3 kPa, *p* < 0.001). A cut-off of 12.5 kPa resulted in AUC, sensitivity and specificity values for predicting AA of 97%, 93% and 100%, respectively [48]. In another study, the 2D-SWE values for the appendix and mesenteric fat were significantly higher in patients with AA than in healthy controls (33.35 ± 11.11 vs. 16.51 ± 3.71 kPa and 33.35 ± 11.9 vs. 16.29 ± 4.2 kPa, respectively, *p* < 0.001). However, appendix diameter measured at conventional US showed an AUC comparable to the 2D-SWE values for distinguishing patients with AA from controls (0.996 vs. 0.998) [49].

Indeed, standard B-mode exhibits a remarkable ability to detect acute inflammatory processes affecting the appendix. In this setting, the real advantage of US-E may lie in its capacity to enhance the diagnostic accuracy of the B-mode when it does not reveal the typical features of appendicitis (such as appendix diameter ≥ 6 mm, wall thickening, echogenicity of periappendiceal adipose tissue, periappendiceal fluid). In a prospective study by Kapoor et al., real-time SE correctly identified all 25 patients with AA, while the B-mode missed three cases [50]. In another study, p-SWE improved traditional US diagnostic efficacy for AA when appendix diameter was inferior to 6 mm [51]. Furthermore, all three negative appendicectomy cases reported in the study by Isik et al., were false positives in B-mode and true negatives in 2D-SWE, considering the optimal cut-off value of 14.5 kPa (AUC 0.93) [52].

Lastly, a relationship between US-E and the severity of AA was demonstrated. A mild/moderate grade of AA assigned by SE demonstrated a high negative predictive value for phlegmonous and perforated appendicitis (99.4 and 100%, respectively) [53].

### 3.3. Neoplastic Bowel Lesions

To the best of our knowledge, only one study analysed the difference in the SE values between colorectal adenocarcinoma and adenomas. In this study, Havre et al., prospectively enrolled patients with scheduled elective bowel resection. The strain ratio measurement and qualitative classification of lesions were performed. Adenocarcinomas were classified in increased tissue hardness categories and showed a strain ratio significantly higher than that in adenomas. However, no significant correlation was found between strain ratio and the grading of adenocarcinomas or in advanced T stages [54].

## 4. Pancreas

**Figure 2:**
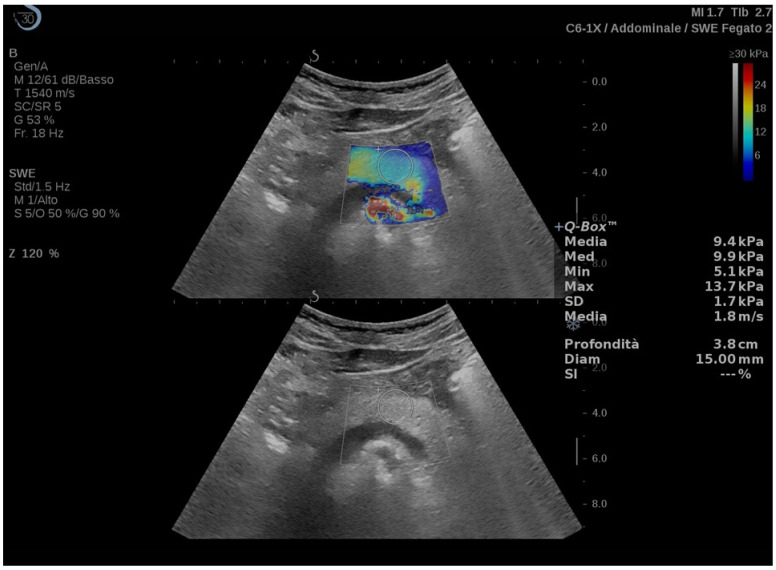
Transverse 2D-shear wave elastography (upper part of the image) and corresponding B-mode (lower part of the image) of the pancreatic body measured with SSI.

Clinical studies regarding the use of US-E in pancreatic diseases are listed in Table 2, while an example of a US-E exam of the pancreas is depicted in Figure 2.

Defining normal elastography values for the pancreatic parenchyma is challenging since, even when using the same method, there is a degree of variation among the healthy control populations presented across the studies. One of the largest healthy cohorts consisting in 210 subjects was presented by Xie et al. Normal pancreatic head and body’s p-SWE values were 1.18 ± 0.23 m/s and 1.20 ± 0.20 m/s, respectively, and were not related to age, gender, BMI, waist circumference and organ dimensions [55]. In contrast, Stumpf et al., reported a lower p-SWE value in men than in women, as well as an the increase with age and a correlation with BMI. Alcohol and tobacco use were not associated with the p-SWE value [56].

**Table 2:** Transabdominal ultrasound elastography (US-E) in pancreatic diseases.

First Author (Year)	Pancreatic Disease	Aim	Study Design (N)	US Device	Elastography Technique	Main Results
Sezgin (2022) [57]	PS	Compare PS to non-PS Correlation with metabolic parameters	Prospective (total 125: PS 68, non-PS 57)	Aplio 500, Toshiba	2D-SWE	2D-SWE was higher in PS than in controls (9.08 ± 2.29 vs. 7.13 ± 1.85, *p* = 0.000). In PS group, 2D-SWE values were significantly correlated with waist circumference (r = 0.335; *p* = 0.023) and insulin resistance (r = 0.338, *p* = 0.004).
Iino (2021) [58]	PC	Differential diagnosis of lesions	Prospective (total 85: PDAC 36, non-PDAC 16, controls 33)	Aplio 500, Canon	2D-SWE	2D-SWE values were higher in PDAC than in non-PDAC (9.8 vs. 7.5 kPa, *p* = 0.0045).
Sezgin (2021) [59]	AP	Compare AP to healthy controls Correlation with AP severity and evolution	Prospective (total 155: AP 81, controls 74)	Aplio 500, Toshiba	2D-SWE	2D-SWE values were higher in AP than in controls (10.97 ± 2.26 vs. 7.72 ± 2.50 kPa, *p* = 0.000) and were not different between mild and severe AP. 2D-SWE values decreased after clinical improvement but remained higher than those in controls after 1 month.
Suzuki (2021) [60]	AIP	Compare AIP to healthy controls Relation with response to therapy	Prospective (total 57: controls 34, AIP 23)	Aplio i900, Canon	2D-SWE	2D-SWE values were higher in AIP than in controls (30.9 vs. 6.6 kPa, *p* < 0.001). Decrease in 2D-SWE values was greater than change in the size of the pancreas (*p* = 0.026).
Sanjeevi (2020) [61]	RAP	Compare RAP to healthy controls	Prospective (total 66: controls 35, RAP 31)	Acuson 2000, Siemens	p-SWE	p-SWE values were higher in RAP than in controls (1.27 ± 0.50 vs. 1.00 ± 0.17 m/s, *p* = 0.001) and correlated with the number of pain episodes.
Durmaz (2018) [62]	AP	Compare AP to CP and healthy controls Diagnose AP	Prospective (total 120: controls 70, AP 50)	Aplio 500, Toshiba	2D-SWE	2D-SWE values were higher in AP than in controls (3.48 ± 0.52 vs. 2.60 ± 1.63 m/s or 45.71 ± 10.72 vs. 23.77 ± 6.72 kPa, *p* < 0.001). The sensitivity and specificity were both 98%, using elastic modulus, and 96% and 98.3%, respectively, using SW velocity.
Kaya (2018) [63]	AP	Compare AP to healthy controls Diagnose AP Correlation with clinical-laboratory outcome	Prospective (total 187: controls 79, AP 108)	Acuson 2000, Siemens	p-SWE	p-SWE values were higher in AP than in controls (2.43 ± 0.08 vs. 1.27 ± 0.025 m/s, *p* < 0.001). The sensitivity was 100%, and the specificity to diagnose AP was 98%. No correlation with hospitalization, amylase and blood leukocyte.
Kuwahara (2018) [64]	CP	Correlation with endoscopic ultrasound findings	Retrospective (85)	iU22, Philips	p-SWE	Hyperechoic foci with shadowing and lobularity with honeycombing were independently related to p-SWE (B = 2.92 (95% CI 2.12–5.71, *p* < 0.001) and B = 3.91 (95% CI 1.22–4.62, *p* = 0.001), respectively).
He (2017) [65]	PS	Compare DM to healthy controls Compare complicated DM to uncomplicated DM	Prospective (total 230: controls 115, complicated DM 68, uncomplicated DM 47)	Acuson S2000, Siemens	p-SWE	p-SWE values were higher in DM than in controls (*p* < 0.001). p-SWE values for pancreatic body were higher in complicated DM than in uncomplicated DM (*p* < 0.01).
Pozzi (2017) [66]	CP	Compare CP to healthy controls Correlation with CP severity	Prospective (total 94: control 42, CP 52)	iU22, Philips	p-SWE	p-SWE values were higher in CP than in controls (4.3 ± 2.4 vs. 2.8 ± 1.1 kPa, *p* = 0.001). Disease duration, analgesics administration and low body weight were independently associated with p-SWE values.
Kuwahara (2016) [67]	-	Correlation with histological pancreatic fibrosis	Retrospective (53)	iU22, Philips	p-SWE	p-SWE values correlated with histological pancreatic fibrosis stage. The AUC for ≥mild, ≥moderate and severe fibrosis was 0.85, 0.84 and 0.87.
Llamoza-Torres (2016) [68]	CP	Diagnose CP	Retrospective (total 33: normal 16, CP 17)	Acuson S2000, Siemens	p-SWE	p-SWE values for pancreatic body were higher in CP compared to normal group (1.57 vs. 1.27 m/s, *p* = 0.037); AUC 0.71 (95% CI 0.532–0.895) to diagnose CP
Xie (2015) [55]	AP	Compare AP to healthy controls	Prospective (total 254: controls 210, AP 44)	Acuson S2000, Siemens	p-SWE	p-SWE showed no differences between AP and healthy controls (1.21 ± 0.20 vs. 1.18 ± 0.20 m/s (pancreatic head) and 1.25 ± 0.19 m/s (pancreatic body)).
Goya (2014) [69]	AP	Compare B-mode, CT and elastography to diagnose AP	Prospective (88)	Acuson S2000, Siemens	p-SWE	p-SWE showed higher accuracy than B-mode and CT to diagnose AP with 100% sensitivity and 98% specificity
Park (2013) [70]	PC	Differential diagnosis benign and malignant lesions	Retrospective (total 27: benign 8, malignant 19)	Acuson S2000, Siemens	p-SWE	Relative p-SWE values were higher for malignancies than for benign lesions (1.5 ± 0.8 vs. 0.4 ± 0.3 m/s, *p* = 0.011)
Mateen (2012) [71]	AP	Compare AP to CP and healthy controls Diagnose AP	Prospective (total 166: controls 52, CP 46, AP 68)	Acuson S2000, Siemens	p-SWE	p-SWE values were higher in AP compared to CP and healthy controls (3.28 ± 0.85 vs. 1.28 ± 0.29 vs. 1.25 ± 0.23 m/s, *p* < 0.001). Sensitivity, specificity, PPV and NPV of p-SWE to differentiate AP vs. CP and healthy controls were 97.1%, 92.9%, 90.4% and 97.8%, respectively.
Yashima (2012) [72]	CP	Compare CP to healthy controls Diagnose CP	Prospective (total 98: controls 52, CP 46)	Acuson S2000, Siemens	p-SWE	p-SWE values for pancreatic head, body and tail were higher in CP than in controls (1.23 ± 0.34, 1.30 ± 0.34 and 1.24 ± 0.50 vs. 1.65 ± 0.71, 2.09 ± 1.03 and 1.68 ± 0.84 m/s, respectively, *p* < 0.001). Considering pancreatic body, AUC was 0.78 with sensitivity, specificity, PPV and NPV of 75%, 72%, 69% and 78%, respectively.

Abbreviations: N, number of patients; AIP, autoimmune pancreatitis; AP, acute pancreatitis; AUC, area under the curve; CP, chronic pancreatitis; CT, computed tomography; DM, diabetes mellitus; NPV, negative predictive value; PC, pancreatic cancer; PDAC, pancreatic ductal adenocarcinoma; PPV, positive predictive value; PS, pancreatic steatosis; p-SWE, point shear eave elastography; RAP, recurrent acute pancreatitis; SW, shear wave; SWE, shear wave elastography.

### 4.1. Acute Pancreatitis

Different studies investigated the ability of US-E to distinguish acute pancreatitis (AP) from normal pancreas or other pancreatic diseases. Mateen et al., found that the p-SWE values were higher in AP patients (*n* = 68) compared to healthy subjects (*n* = 52) and CP patients (*n* = 46) (3.28 ± 0.85 vs. 1.28 ± 0.29 and 1.25 ± 0.24 m/s, *p* < 0.001). Using a cut-off value of 2.2 m/s, the negative predictive value of p-SWE to distinguish AP from healthy controls and CP patients was 97.8%. Interestingly, five patients with AP had the p-SWE values higher than the upper detection limit, but different grey scale and colour scale scores, suggesting that the combination of two elastography techniques could improve the distinction between the inflamed tissue and tissue necrosis [71]. In contrast, Xie et al., found no significant difference between patients with AP (*n* = 44) and healthy controls in the p-SWE results [55]. Given that PA is usually diagnosed based on clinical and laboratory findings in typical cases, US-E could improve the diagnostic accuracy in unclear cases, even when imaging is inconclusive. Using a cut-off value of 1.63 m/s, the p-SWE showed better performance than computerized tomography (CT) in diagnosing AP (diagnosed based on clinical and laboratory findings), with a sensitivity and specificity of 100% and 98%, respectively [69]. Similarly, Durmaz et al., showed that in clinically and laboratory-diagnosed AP patients with normal US and normal contrast-enhanced CT, the 2D-SWE values were significantly higher than those in asymptomatic volunteers (41.67 ± 9.65 vs. 23.77 ± 6.72 kPa, *p* < 0.001) [62].

Given that universal normal or pathological elastography values have not been clearly determined, it would be highly interesting to investigate the relationship between the change in elastography values between the initial time and subsequent follow-up and prognostic outcomes. In a prospective study classifying AP according to Atlanta criteria, the 2D-SWE values in patients with mild AP trended to be higher than those in patients with severe AP (9.05 ± 1.44 vs. 8.61 ± 1.72 kPa); however, the difference was not significant. After clinical improvement, the 2D-SWE values decreased considerably compared to the initial value (10.97 vs. 8.8 kPa, *p* = 0.000) but remained elevated after 1 month. Furthermore, 2D-SWE was not correlated with hospitalization and biochemical parameters [59]. Confirming this, Kaya et al., showed no correlation between the p-SWE values and the length of hospitalization, amylase and white blood cell count. Furthermore, the p-SWE value was not different between patients who developed complication and those who did not (2.70 ± 0.247 vs. 2.36 ± 0.63, *p* = 0.151) [63]. Further studies to evaluate the variation of the SWE values during the AP evolution are clearly warranted.

Another important issue is to clarify if US-E can early detect the evolution of AP in CP patients. In patients with idiopathic recurrent AP and without endoscopic ultrasound (EUS) criteria of CP, the mean p-SWE value was significantly higher compared to that of healthy controls (1.27 ± 0.50 vs. 1.00 ± 0.17 m/s, *p* = 0.001) and showed a positive correlation with the number of pain episodes, suggesting that fibrosis increases after each episode [61].

The US-E could be useful also to monitor response to therapy. In patients with type 1 autoimmune pancreatitis (*n* = 23), the 2D-SWE values were significantly higher than those in healthy controls (*n* = 34) (30.9 vs. 6.6 kPa, *p* = 0.001) and decreased over two weeks of steroids therapy more than the change in the pancreatic parenchyma size, proving to be a sensitive tool in monitoring response to therapy. Interestingly, the SW dispersion slope, which reflects the viscosity of a tissue and is related to necroinflammation more than fibrosis, was significantly different between patients with autoimmune pancreatitis and controls (15.3, IQR 4.2 vs. 13, IQR 3.4, *p* = 0.011) [60].

### 4.2. Chronic Pancreatitis

CP is characterised by the progressive replacement of pancreatic parenchyma with fibrotic tissue and the potential of progression to exocrine and endocrine insufficiency [73]. As far as we are aware, only one study has histologically demonstrated the relation between fibrosis and the US-E findings. In fifty-three patients undergoing surgical pancreatic procedures for different indications (mainly malignancies and cystic lesion), preoperative p-SWE and histological analysis of the same region were performed. The p-SWE results demonstrated a significant positive correlation with the fibrosis stage and an AUC of 0.85, 0.84, and 0.87 for differentiating between mild or higher, moderate or higher and severe fibrosis, respectively [67].

Regarding the diagnostic value of US-E in CP, in a prospective study including patients with suspected CP (diagnosed with EUS), the p-SWE of the pancreatic body had significantly higher results in patients with the confirmed diagnosis of CP compared to patients without CP (1.57 vs. 1.27 m/s, *p* = 0.037), with an AUC of 0.71 for diagnosed CP. Furthermore, the p-SWE value correlated with the number of the EUS criteria for CP [68]. Similarly, Kuwahara et al., showed a correlation between the p-SWE values, Rosemont classification, Japan Pancreas Society clinical diagnostic criteria 2009 and the numbers of the EUS criteria for CP, obtaining an AUC of 0.77 for definitive or suggestive CP diagnosis. At multivariate regression, hyperechoic foci with shadowing and lobularity with honeycombing were independently related to the p-SWE values [64]. Compared to Llamoza-Torres et al.’s study, Yashima et al., described significantly higher values of p-SWE in each part of the pancreas (head, body and tail) compared to those in healthy volunteers (1.65 ± 0.71, 2.09 ± 1.03, 1.68 ± 0.84 m/s vs. 1.23 ± 0.34, 1.30 ± 0.34 and 1.24 ± 0.50 m/s, respectively, *p* < 0.001) [72]. Notably, 74% of CP cases in this study were categorised as Cambridge grade V, and 76% of patients had alcoholic CP, indicating that the SWE values may increase as CP progresses and may be influenced by the CP aetiology. Supporting this, the p-SWE values were significantly higher in the CP patients with the disease duration > 10 years, who required chronic analgesics, and who had a lower body weight, and all of the results were independently associated with the p-SWE values [66]. Further studies are required to confirm the association between the progression of CP and changes in the US-E findings and to investigate the role of elastography in predicting exocrine pancreatic insufficiency.

### 4.3. Metabolic Associated Pancreatic Disease

Pancreatic steatosis (PS) is an emerging pathological entity that can contribute to the development and progression of pancreatic inflammatory and neoplastic disease, as well as endocrine systemic alteration and metabolic disorders [74]. Therefore, PS, non-alcoholic fatty pancreas disease (NAFPD) and non-alcoholic fatty steatopancreatitis are the terms used to defined specific conditions [75].

As triglyceride accumulation modifies the viscoelastic properties of tissue, US-E may reflect these alterations; however, a comparison with histological data is not easily achievable. Recently, Sezgin et al., showed that the 2D-SWE values were significantly higher in PS patients compared to non-PS group (9.08 ± 2.29 vs. 7.13 ± 1.85 kPa, *p* = 0.000). In addition, the 2D-SWE values correlated with BMI, waist circumference and serum triglycerides, progressively increased in B-mode classes of PS, and were significantly higher in patients with metabolic syndrome [57]. These results suggest that the stiffness of the pancreas increases with the PS grade and reflects the impairment of the pancreatic function and the progression of metabolic disorder. Supporting this, the p-SWE values for the pancreas body were significantly higher in patient with diabetes mellitus and microangiopathy compared to those in patients with uncomplicated diabetes (*p* < 0.01) [65].

### 4.4. Pancreatic Cancer

Although the US-E has been shown to improve the diagnostic accuracy of the B-mode in determining the precise type of pancreatic lesions [76], contradictory evidence exists regarding the validity of the absolute SWE value in discriminating pancreatic lesions. Some authors showed a significantly higher SWE value in tumour site compared to normal pancreas [77]. Other studies indicates that absolute SWE values of pancreatic cancer and normal parenchyma [78] or benign lesions and malignant ones are not significantly different [70]. On the other hand, relative SWE values provide greater potential for mass differentiation. Park et al., reported that the difference in the mean p-SWE values between lesion and adjacent parenchyma (relative p-SWE value) was significantly lower for benign lesions (mass forming pancreatitis and autoimmune pancreatitis) than for malignant lesions (pancreatic adenocarcinoma (PDAC), neuroendocrine malignant tumour, metastasis) (0.4 ± 0.3 vs. 1.5 ± 0.8 m/s *p* = 0.011) [70]. Likewise, the 2D-SWE tumour/parenchyma ratio > 2.42 kPa in histologically defined pancreatic lesions demonstrated an AUC of 0.77 for distinguishing PDAC from non-PDAC [58].

The US-E may also have a prognostic role in pancreatic cancer. Preliminary results of the study conducted by Kawada et al., demonstrated that strain ratio modification was related to response to therapy [79]. Further research is needed to establish the real diagnostic and prognostic role of quantitative US-E in pancreatic cancer.

## 5. Gallbladder

### 5.1. Inflammatory Diseases

By using conventional US, acute cholecystitis is diagnosed with 81% sensitivity and 80% specificity [80]. When classical B-mode US features of acute cholecystitis (presence of gallstone, sonographic Murphy sign, gallbladder wall thickening) are not detected, the diagnosis could be challenging. Can US-E enhance the diagnostic efficacy of B-mode in the diagnosis of acute cholecystitis and how? US-E could provide additional information to conventional B-mode findings by analysing the change in elasticity of pericholecystic liver parenchyma caused by pathophysiological changes in acute cholecystitis. It was demonstrated that the 2D-SWE values for the gallbladder bed of the liver were significantly higher in patients with acute cholecystitis compared to patients with non-acute cholecystitis (8.2 vs. 5.8 kPa, *p* = 0.045). A cut-off value of 8 kPa showed an AUC of 0.69 with high specificity (90%) and low sensitivity (57.1%) and categorized the 2D-SWE value as the only factor significantly related to acute cholecystitis [81]. Similarly, Kim et al., demonstrated that the addition of p-SWE findings to conventional US significantly enhanced the AUC of two examiners in the diagnosis of acute cholecystitis from 0.79 and 0.78 to 0.96, respectively [82].

### 5.2. Benign and Malignant Lesions

US-E could be an additional tool to distinguish malignancies from benign condition of gallbladder wall. Using SE, gallbladder polyps were characterized by medium-high strain (soft or moderately soft) compared to only one single small nodule (measuring 19 mm) defined as gallbladder carcinoma which exhibited low strain (high stiffness) [83]. Considering differential diagnosis between benign wall thickening and gallbladder carcinoma, Kapoor et al., reported that the p-SWE values could distinguish between the two conditions with 92.8% accuracy [84]. Furthermore, the 2D-SWE showed an AUC of 0.92 to differentiate gallbladder carcinoma from chronic cholecystitis, resulting in a significantly lower value for chronic cholecystitis compared to gallbladder carcinoma and apparently non-involved wall in gallbladder carcinoma (12.3 vs. 35.0 and vs. 18.3 kPa, respectively, *p* = 0.01) [85].

## 6. Conclusions

In this review, we examined the numerous applications of elastography in a wide range of gastrointestinal disorders other than the well-established use in liver diseases. Elastography could be a useful adjunct to conventional US in an emergency setting. Elastography has the potential to be an efficient non-invasive diagnostic and characterization tool for acute and chronic conditions of the bowel, pancreas and gallbladder, and finally, it could be an easily accessible and low-cost tool for assessing or monitoring the response to specific treatments. On the other hand, the limitations of current research and the resulting inability to provide detailed indications from international guidelines prevent the application of elastography in routine clinical practise. Additional research is required to standardize the methodology of this technique and to understand the real importance of US-E in all the illustrated conditions.

## Figures and Tables

**Table 1:** Transabdominal ultrasound elastography (US-E) in inflammatory bowel disease (IBD).

First Author (Year)	IBD	Aim	Study Design (N)	US Device	Elastography Technique	Main Results	**Reference Method**
Abu Ata (2023) [23]	CD	Stricture characterization	Prospective (18)	Acuson S3000, Siemens	2D-SWE	2D-SWE values negatively correlated with mucosal inflammation (r = −0.50, *p* = 0.04) and positively correlated with muscularis propria inner layer smooth muscle hypertrophy (r = 0.72, *p* = 0.002). No correlation was found between 2D-SWE values and fibrosis.	Histology
Chen (2022) [24]	CD	Response to therapy	Prospective (30)	Aixplorer, SuperSonic Imagine	2D-SWE	2D-SWE values decreased in responding patients two weeks after starting therapy (15.3 ± 4.7 vs. 12.6 ± 3.3 kPa, *p* = 0.003). At baseline, 2D-SWE values were higher in non-responders’ group (15.3 ± 4.7 vs. 21.3 ± 8.7 kPa, *p* = 0.022).	Clinical data and endoscopy
Yamada (2021) [25]	UC	Disease activity	Cross-sectional (26)	Aplio i900, Canon	2D-SWE	2D-SWE values were higher in mucosal healing group compared to active disease group (2.40 (IQR, 2.18–3.38) vs. 1.62 (IQR, 1.44–1.95) m/s, *p* = 0.007).	Endoscopy
Ma (2020) [26]	CD/UC	Disease activity	Retrospective (30)	Logiq E8, GE	2D-SWE	SWE values were higher in fibrosis group compared to inflammation group (3.63 ± 0.86 vs. 2.51 ± 0.66 m/s, *p* = 0.004).	CEUS and traditional US
Ding (2019) [27]	CD	Stricture characterization	Prospective (25)	Acuson S2000, Siemens	SE (colour scale classification) p-SWE	p-SWE values were higher in fibrotic strictures compared to inflammatory stenosis (1.57 ± 0.60 vs. 2.59 ± 0.97 m/s, *p* < 0.05) and superior to SE in differentiating stenosis properties.	Histology
Goertz (2019) [28]	UC	Disease activity	Prospective (20)	Acuson S2000, Siemens	p-SWE	p-SWE values were not correlated with MAYO subscore or C-reactive protein level.	Clinical and laboratory data
Chen (2018) [29]	CD	Stricture characterization	Prospective (35)	Aixplorer, SuperSonic Imagine	2D-SWE	2D-SWE values were higher in severe fibrosis compared to mild or moderate fibrosis (23.0 ± 6.3 vs. 17.4 ± 3.8 and 14.4 ± 2.1 kPa, *p* = 0.008).	Histology
Goertz (2018) [30]	CD	Disease activity	Retrospective (77) Prospective (21)	Acuson S2000, Siemens	p-SWE	No significant correlation was found between ileal or sigma’s p-SWE values and disease activity indicators.	Clinical and laboratory data
Orlando (2018) [31]	CD	Response to therapy	Prospective (30)	iU22, Philips	SE (SR)	SR values at baseline were lower in patients who achieved mucosal healing after 14 weeks compared to patients not achieving this endpoint (1.06 vs. 1.58, *p* = 0.03).	Traditional US (mucosal healing = bowel wall thickness < 3 mm)
Quaia (2018) [32]	CD	Stricture characterization	Prospective (20)	iU22, Philips	SE (colour scale classification)	Combination of B-mode, CEUS and SE showed higher diagnostic accuracy than each single technique.	Histology
Lu (2017) [33]	CD	Stricture characterization	Prospective (105 CEUS/15 histology)	Acuson S3000, Siemens or Epiq 5, Philips	p-SWE	p-SWE values moderately correlated with muscular hypertrophy (r = 0.59, *p* = 0.02) and negatively correlated with peak enhancement (r = −0.59, *p* = 0.02). No correlation was found with fibrosis or inflammation.	Histology
Serra (2017) [34]	CD	Stricture characterization	Prospective (32)	iU22, Philips	SE (SR)	No correlation was found between SR and inflammatory and fibrosis scores.	Histology
Sconfienza (2016) [35]	CD	Stricture characterization	Prospective (16)	MyLab 70 XvG, Esaote	SE (colour scale score)	SE score was lower in inflammatory strictures compared to fibrotic strictures (16 (IQR 16–18) vs. 20 (IQR, 17.5–22), *p* = 0.003).	Magnetic resonance elastography
Fraquelli (2015) [36]	CD	Stricture characterization	Prospective (23)	iU22, Philips	SE (colour scale classification and SR)	SR values were associated with ileal fibrosis at multivariate analysis (R^2^ = 0.75, *p* < 0.0001). No significant association was found between colour scale values and fibrosis.	Histology
Fufezan (2015) [37]	CD	Disease activity	Prospective (14)	Xario V 2.0 (Toshiba)	SE (colour scale classification) and SR)	Colour classes and SR results were significantly associated with disease activity markers (CRP-SE *p* = 0.0104, CRP-SR *p* = 0.0721, ESR-SE *p* = 0.0008, ESR-SR *p* = 0.0123, CAL-SE *p* =0.0139).	Laboratory data
Ishikawa (2011) [38]	UC	Disease activity	Prospective (37)	EUB-8500, Hitachi	SE (colour scale classification)	Elastography and colonoscopy findings were significantly associated (*p* < 0.001).	Endoscopy

Abbreviations: IBD, inflammatory bowel disease; N, number of patients; US, ultrasound; CD, Crohn’s disease; CAL, calprotectin; CRP, C-reactive protein, ESR, erythrocyte sedimentation rate; p-SWE, point-shear wave elastography; CEUS, contrast-enhanced ultrasound; SE, strain elastography; SR, strain ratio; SWE, shear wave elastography; UC, ulcerative colitis.

## Data Availability

Data sharing not applicable.
